# Berberine chloride can ameliorate the spatial memory impairment and increase the expression of interleukin-1beta and inducible nitric oxide synthase in the rat model of Alzheimer's disease

**DOI:** 10.1186/1471-2202-7-78

**Published:** 2006-12-01

**Authors:** Feiqi Zhu, Caiyun Qian

**Affiliations:** 1 The neurology department of the first affiliated hospital, Sun Yet-Sen University, Guangzhou, Guangdong province, 510080, PR China

## Abstract

**Background:**

Berberine is the major alkaloidal component of *Rhizoma coptidis*, and has multiple pharmacological effects including inhibiting acetylcholinesterase, reducing cholesterol and glucose, lowering mortality in patients with chronic congestive heart failure and anti-inflammation etc. Thus berberine is a promising drug for diabetes, hyperlipemia, coronary artery disease and ischemic stroke etc. The present study was carried out to investigate the effect of berberine chloride on the spatial memory, inflammation factors interleukin-1 beta (IL-1beta) and inducible nitric oxide synthase (iNOS) expression in the rat model of Alzheimer's disease (AD) which was established by injecting Abeta (1–40) (5 microgram) into the rats hippocampuses bilaterally.

**Results:**

The rats were given berberine chloride (50 mg/kg) by intragastric administration once daily for 14 days. The spatial memory was assayed by Morris water maze test, IL-1beta and iNOS in the hippocampus were assayed by immunohistochemistry and real time polymerase chain reaction (PCR). Intragastric administration of berberine significantly ameliorated the spatial memory impairment and increased the expression of IL-1beta, iNOS in the rat model of AD.

**Conclusion:**

Berberine might be beneficial to AD by intragastric administration though it might exaggerate the inflammation reaction.

## Background

AD is the most prominent dementia in senile population affecting approximately 5% of the over 65-year old populations, but the cause of AD remains largely unknown. In the pathogenesis of AD, the inflammation mechanism is seemed to play an important role [1]. The senile plaque is the hallmark of AD. The core of the senile plaque is the deposition of β-amyloid (Aβ) and the activated microglia and astroglia are around the senile plaque. In these glias, numerous inflammation factors including IL-1β, interleukin -6(IL-6), tumor necrosis factor-α (TNF-α) and iNOS etc, are overexpressed. These inflammation factors have been seemed to be neurotoxic. At the same time, some epidemiological studies demonstrated that long-term use of nonsteroidal anti-inflammatory drugs (NSAIDs) could prominently delay the onset of AD [2-5]. The current drugs for AD treatment including cholinesterase inhibitors (donepezil, rivastigmine and galanthamin) and N-methyl-D-aspartate (NMDA) receptor antagonist (memantine) which were approved by Food and Drug Administration of USA (FDA) are symptomatic treatment, but these drugs usually can not delay the development of AD. Anti-inflammation drugs are expected to delay AD, but most clinical research results of NSAIDs on AD are negative [6]. Moreover some selective COX-2 inhibitors increased the occurrence of cardiovascular accidents [7,8].

Berberine is an isoquinoline alkaloid with a long history of medicinal usage in China. It exists in *Hydrastis canadensis *(goldenseal), *Cortex phellodendri *(Huangbai) and *Rhizoma coptidis *(Huanglian). These medicinal plants have been widely used as traditional medicines for treating diarrhea and gastrointestinal disorders for a long time in China. Berberine, the major ingredient of these herbs, has multiple pharmacological effects. It is a acetylcholinesterase inhibitor similar to Galanthamine[9], a drug treating AD, and might be a low-molecular-weight neurotrophic drug to neurodegeneration disorder such as AD by potentiating the nerve growth factor (NGF)-induced differentiation in neural cells [10]; berberine is a novel cholesterol-lowering drug distinctly from statins by stabilizating the low density lipoprotein receptor (LDLR) mRNA to increase LDLR expression and inhibiting lipid synthesis[11,12]. Berberine is also able to exert a glucose-lowering effect by insulin independent manner [13], stimulating insulin secretion and effectively sensitizing insulin activity [14,15]. So berberine can play an important role on metabolic syndrome. Berberine could improve quality of life and decrease ventricular premature complexes and mortality in patients with chronic congestive heart failure [16]. Berberine has potential in the prevention of atherosclerosis and restenosis [17,18]. Berberine can block transient outward potassium current (*IA*) and delayed rectifier potassium current (*IK*) in a concentration-dependent manner in acutely isolated CA1 pyramidal neurons of rat hippocampus [19]. A recent study demonstrated that mutations in voltage-gated potassium channel KCNC3 caused adult-onset ataxia [20], and potassium channels are regarded to play a key role in neurodegeneration including AD [21]. So berberine might be useful for the treatment of neurodegeneration disorders including AD.

Except the above pharmacological effects, extracts obtained from the roots of berberidaceae species have been used in Eastern and Bulgarian folk medicines for the treatment of rheumatic and other chronic inflammatory disorders. Berberine suppressed a delayed type hypersensitivity (DTH) reaction in a chronic inflammatory model of adjuvant arthritis and diminished the antibody response against sensitization red blood cell (SRBC) *in vitro *[22]. In recent years, it was demonstrated that berberine could inhibit the expression of some inflammation factors. It might inhibit arachidonic acid metabolism in rabbit platelets and endothelial cells [23]. Berberine could decrease cyclooxygenase-2 (COX-2) expression by directly inhibiting the activator protein-1(AP-1) binding and IL-1β and TNF-α productions in HepG2 cells and cardiomyocyte through nuclear factor-κB (NF-κB) signaling pathway [24-27]. It could decrease IL-6 production in esophageal cancer cells and inhibit expression of iNOS mRNA in ethanol-induced gastric ulcer mice [28,29].

So berberine might be a very promising drug to treat the cardiac disease, stroke, diabetes and hyperlipoidemia and chronic inflammation diseases. So far, whether berberine might be beneficial to AD by decreasing the inflammation factors expression has not been studied. To test the hypothesis that berberine might inhibit the inflammation factors IL-1β and iNOS expression in the rat model of AD, we used the rat model of AD established by injecting Aβ(1–40)(5 μg) into the bilateral hippocampuses with stereotaxic coordinates and gave the rats berberine chloride (50 mg/kg) intragastricly for 14 days. It was very surprising that we found berberine chloride could significantly ameliorate the spatial memory impairment and increase these two factors expression.

## Results

### Effects of berberine chloride on the impairment of spatial memory

**Figure 1:**
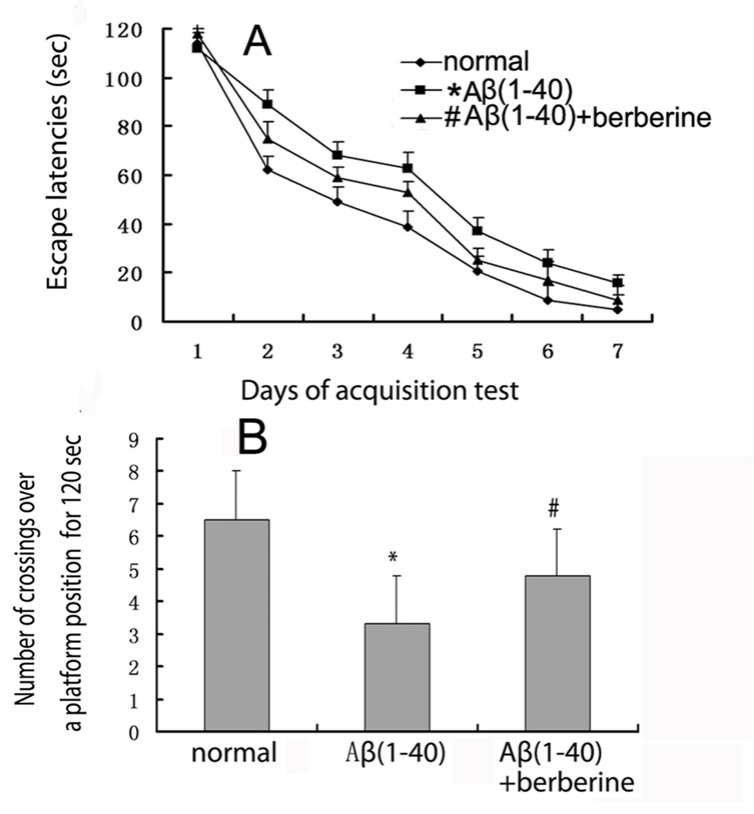
**Effect of berberine chloride on the impairment of spatial memory induced by Aβ(1–40) injection**. (A) Escape latencies per group of four trials tested in a morris water maze over 7 days. (B) The number of crossings over a position where a platform had been was measured for 120 s at seventh days after the last acquisition test. Values represent the means ± SD of 6 rats. * P < 0.01 when compared with the normal group. # P < 0.01 when compared with the Aβ(1–40) injection group.

Rats were trained in the water maze for 7 days starting at the eighth day after the injection of Aβ(1–40) (5 μg). Rats were more efficient at finding the platform on successive trails (Figure 1A). The main effect for each day was significant (P < 0.01). The main effect of berberine chloride was also significant (P < 0.01). Two-way repeated measure analysis of variance (two-way RM ANOVA) revealed a significant increase in escape latency to find the platform in the Aβ(1–40)-injected group as compared with the normal group (P < 0.01) and a significant decrease in escape latency when berberine chloride (50 mg/kg) was intragasticly administrated as compared with the Aβ(1–40) -injected group(P < 0.01).

In the retention test (Figure 1B), the number of crossings over a platform position was significantly decreased in the Aβ(1–40)-injected group as compared with that in the normal group (P < 0.01). The crossing number was recovered by treatment with berberine chloride and the recovery was significant (P < 0.01). All rats showed normal swimming performance and constant increases in body weight. Locomotor activity did not show difference among groups.

### Immunohistochemical evaluation of the effects of berberine chloride on the expression of IL-1β and iNOS

**Figure 2:**
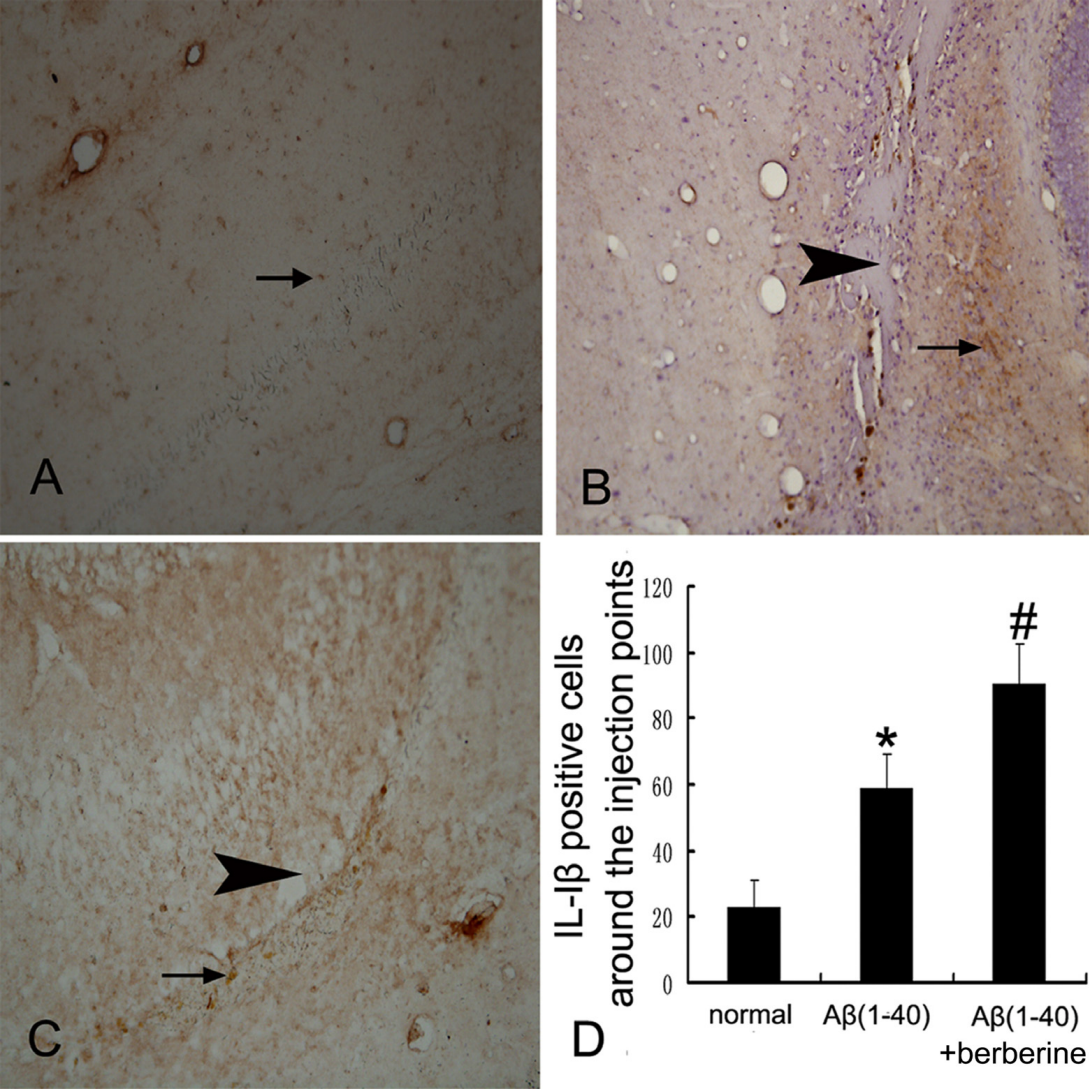
**Immunohistochemistrical evaluation of the effect of berberine chloride on the expression of IL-1β**. (A) the expression of IL-1β(arrow) in the normal rat hippocampus.(B) the expression of IL-1β(arrow) around the injection point (arrowhead) in the Aβ(1–40) group. (C) the expression of IL-1β(arrow) around the injection point (arrowhead) in the Aβ(1–40)+berberine group.(D) the table represents the number of IL-1β positive cells around the injection point in 400× fields. Values represent the means ± SD of 6 rats.*P < 0.01 when compared with the normal group. # P < 0.01 when compared with the Aβ(1–40) group.(A,B,C: 200×)

**Figure 3:**
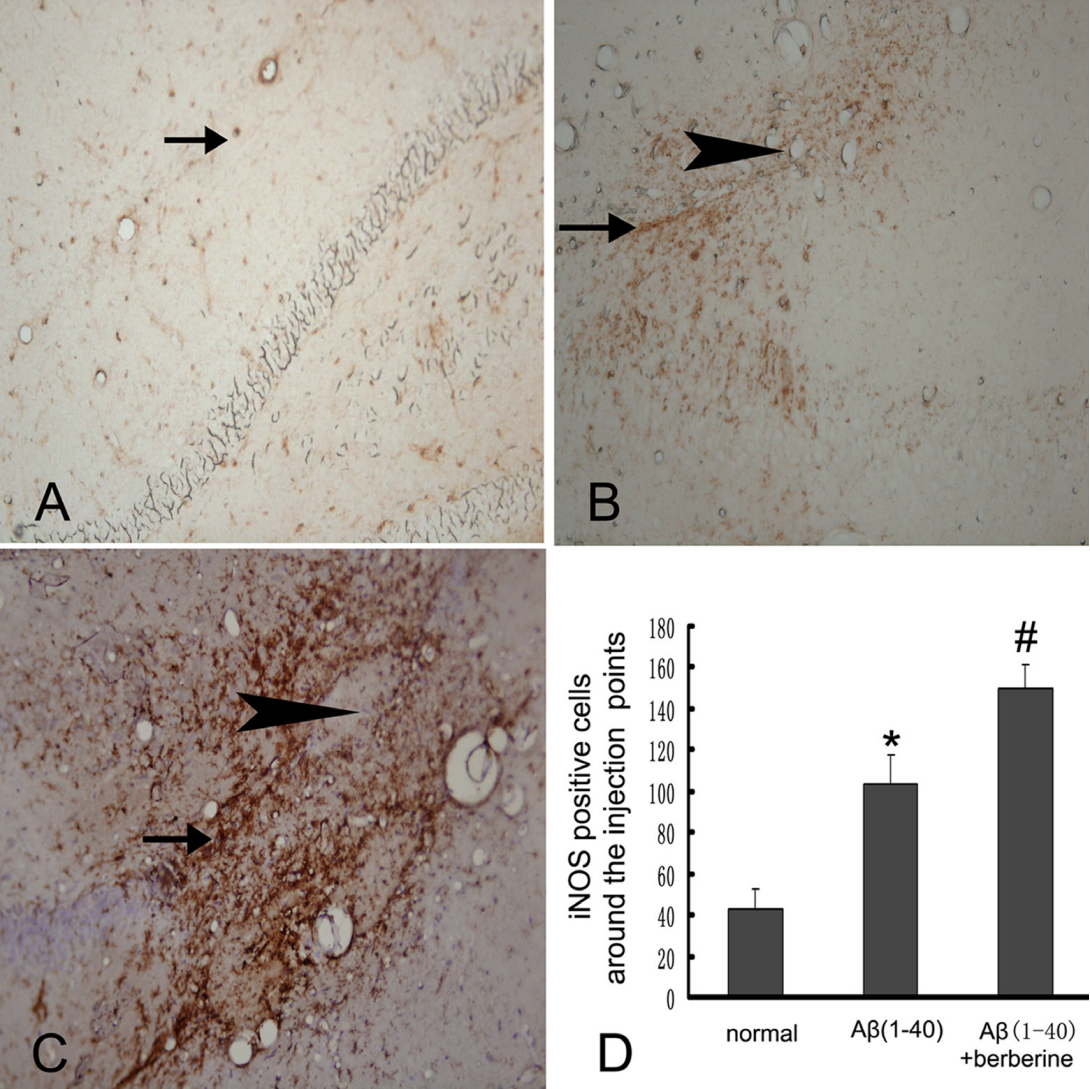
**Immunohistochemistrical evaluation of the effect of berberine chloride on the expression of iNOS**. (A) the expression of iNOS(arrow) in the normal rat hippocampus. (B) the expression of iNOS(arrow) around the injection point (arrowhead) in the Aβ(1–40) group. (C) the expression iNOS (arrow) of around the injection point (arrowhead) in the Aβ(1–40)+berberine group.(D) the table represents the number of iNOS positive cells around the injection point in 400× fields. Values represent the means ± SD of 6 rats. *P < 0.01 when compared with the normal group. # P < 0.01 when compared with the Aβ(1–40) group.(A,B,C: 200×)

The effects of berberine chloride on the expression of IL-1β and iNOS were investigated after the berberine chloride treatment (50 mg/kg) for 14 days by immunohistochemistry. The injection of Aβ(1–40)(5 μg) in the hippocampus highly induced the increase of IL-1β and iNOS immunostainings (P < 0.01), which were more significantly increased in the berberine chloride-treated rats(P < 0.01) (Figure 2, 3). The number of IL-1β-positive cells increased from 22.7 ± 8.3 (normal group) to 58.8 ± 10.7 (Aβ(1–40) group) and 90.5 ± 12.2 (Aβ(1–40) + berberine group) (Figure 2D), and the number of iNOS-positive cells also increased from 42.5 ± 10.2 (normal group) to 103.0 ± 13.9 (Aβ(1–40)group) and 149.5 ± 11.9 (Aβ(1–40) + berberine group) (Figure 3D).

### Evaluation of the effects of berberine chloride on the mRNA of IL-1β and iNOS by real time PCR

**Figure 4:**
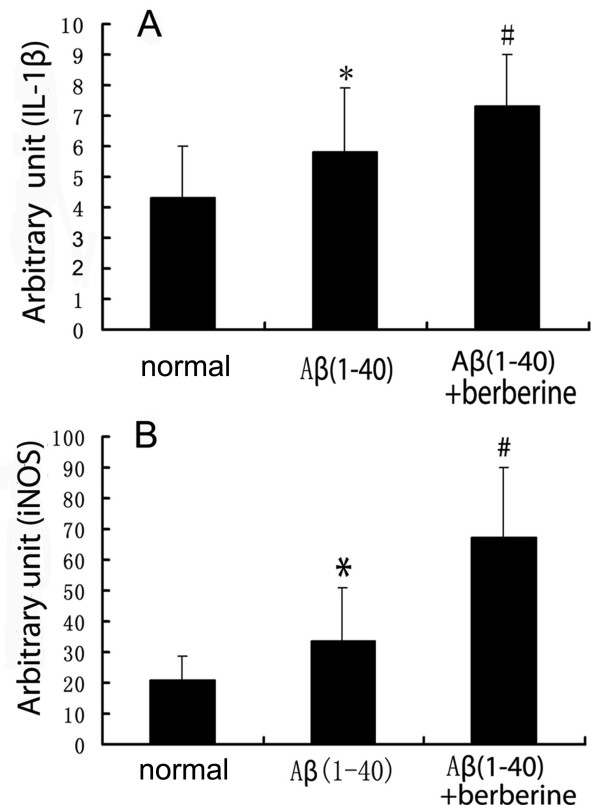
**Evaluation of the effects of berberine chloride on the mRNA of IL-1β and iNOS by Real time PCR**. (A)Real time PCR results of berberine chloride on the IL-1β mRNA indicate that IL-1β mRNA level was increased in the Aβ(1–40) group and Aβ(1–40)+berberine group. The result represents at least three independent experiments, and β-actin was used as an internal control.(B) Real time PCR results of berberine chloride on the iNOS mRNA indicates that iNOS mRNA level was increased in the Aβ(1–40) group and Aβ(1–40)+berberine group. The result represents at least three independent experiments, and β-actin was used as an internal control. Values represent the means ± SD of 6 rats. *P < 0.01 when compared with the normal group. # P < 0.01 when compared with the Aβ(1–40) injection group.

The effects of berberine chloride on the mRNA of IL-1β and iNOS were investigated after berberine chloride (50 m/kg) treatment for 14 days by real time PCR. Injection of Aβ(1–40) (5 μg) in the hippocampus highly increase the mRNA of IL-1β and iNOS (P < 0.01), which were even more significantly increased in the berberine chloride-treated rats (P < 0.01). The relative copies of IL-1β mRNA on internal control β-actin (arbitrary unit) in the Aβ(1–40) group and Aβ(1–40) + berberine group were increased from 4.3 ± 1.7 to 5.8 ± 2.1 and 7.3 ± 1.7, respectively (Figure 4A), and the relative copies of iNOS mRNA on internal control β-actin (arbitrary unit) in the above two groups were increased from 20.7 ± 7.9 to 33.7 ± 17.4 and 67.5 ± 22.5, respectively (Figure 4B).

## Discussion

In this study, we observed that berberine chloride (50 mg/kg) could significantly decrease the escape latency and increase the number of crossings over the platform position in the rats model of AD assayed by Morris water maze test, and could also increase both IL-1β and iNOS expression very significantly assayed by immunohistochemical method. These results are not coincide with the previous studies that berberine could inhibit the expression of iNOS and IL-1β [26,29]. The difference between our results and those of previous studies might be due to the different administration mode of berberine and the effect that berberine can increase non-specific immunity. Berberine was intraperitoneally administrated or used in cells culture model in the reported studies in contrast to the intragastric administration in the present experiment. The uptake rate of berberine by intragastric administration is very low [30]. So the concentration of berberine in blood or cell culture medium in the reported studies is higher than the level in the present investigation. The different level of berberine may cause different effects on the immunity and inflammation factors' expression.

Berberine has been used by oral administration as an unprescribed drug in China, and the safety and efficacy of berberine by this administration mode have been generally accepted. Moreover, berberine (50 mg/kg -100 mg/kg) by intragastric administration can significantly exert cholesterol-lowering effect on rats [11]. So in this study, we chosed the dose 50 mg/kg to study berberine's effect on the expression of IL-1β and iNOS because this dose and administration mode are more close to the clinical practice than veno-injection or intraperitoneal administration. We presume that the increase of inflammation factors IL-1β and iNOS might be related to the rising of microglia activation. In another two previous studies, berberine was demonstrated to be able to activate macrophages and increase the phagcytosing function of peritoneal macrophages (PMΦ) and the production of IL-1 by PMΦ in mice [31,32]. It is indicated that berberine is a potent macrophage activator and can increase non-specific immunity. Moreover it was demonstrated that berberine could decrease the expression of PPARγ_2 _mRNA and protein which was a negative regulator of inflammation[33,34]. Microglia and PMΦ are originated from the peripheric mononuclear macrophage. So berberine might be able to activate the microglia to phagocytize the exogenous Aβ(1–40) which was injected into hippocampus. In another our study, we had also demonstrated that berberine was able to increase the microglia activation and significantly decrease the expression PPAR_γ _after the injection of Aβ(1–40) into the hippocampus (data not shown). So in this study, berberine should be able to increase the inflammation factors expression including IL-1β and iNOS by increasing microglia activation.

It seems that berberine might not be beneficial to AD because it can increase the inflammation factors' expression and microglia activation. But inflammation factors might also be beneficial to central nervous system (CNS). It was reported that IL-1β might be crucial to the repair of the CNS presumably through the induction of astrocyte and microglia-macrophage-derived insulin-like growth factor-1(IGF-1), and IL-1β^--/- ^mice failed to remyelinate properly which was correlated to the lack of IGF-1 production by microglia-macrophages and astrocytes and to a profound delay of precursors differentiating into mature oligodendrocytes [35]. So berberine might ameliorate the pathologies of AD through increasing the expression of IL-1β. At the same time, a very recent study demonstrated that genetic removal of iNOS in mice expressing mutated amyloid precursor protein resulted in pathological hyperphosphorylation of mouse tau, its redistribution to the somatodendritic compartment in cortical and hippocampal neurons, and aggregate formation. Lack of iNOS in the amyloid precursor protein Swedish mutant mouse would increase the insoluble Aβ levels, neuronal degeneration, caspase-3 activation and tau cleavage. This phenomenon suggested that nitric oxide (NO) acted as a junction point between Aβ, caspase activation, and tau aggregation, and iNOS deletion could promote multiple pathologies in a mouse model of AD [36]. So berberine might be able to ameliorate the multiple pathologies of AD by increasing iNOS expression. Moreover, berberine might be beneficial to the senile plaque clearance by increasing microglia activation. A recent study demonstrated that bone marrow-derived microglia played a critical role in restricting senile plaque formation in AD, and therapeutic strategies aiming to improve bone marrow-derived microglia recruitment could potentially lead to a new powerful tool for the elimination of toxic senile plaques [37]. Another recent study also demonstrated that suppressing microglia activation and reducing inflammation factors expression by minocycline could enhance senile plaque formation [38]. Moreover AD might be associated with the aging of microglia for postnatal microglia, non-adult microglia has the ability to phagocytose Aβ fibrils[39]. The above studies indicated that anti-inflammation drugs might not be beneficial to AD, which is consistent with the vague effect of this kind of drugs including NSAIDs on AD. So berberine might be beneficial to AD by enhancing the bone barrow derived mononuclear macrophage to cross the blood-brain barrier (BBB) and to be activated microglia which has the ability to phagocytose Aβ fibrils, clear the senile plaque and produce the more inflammation factors.

## Conclusion

Berberine might be beneficial to AD by ameliorating spatial memory impairment, improving inflammation factors expression, activating microglia and the senile plaque clearance. But further investigation is needed in AD transgenic models and clinical research to demonstrate the efficacy of berberine to AD.

## Methods

### Animals and drug treatments

Adult male Sprague-Dawley rats (n = 18) (220–250 g, experimental animal center of Sun Yet-Sen University) were housed 6 per cage with free access to food and water, and were kept in a constant environment (22 ± 2°C, 50 ± 5% humidity, 12-h light/dark cycle). The rats were randomly divided into three groups, including the normal group, the Aβ(1–40) group and Aβ(1–40) + berberine group. Aβ(1–40) (Sigma, St. Louis, MO.USA) was dissolved in sterile distilled water at a concentration of 5 μg/μL, and incubated at 37°C for 7 days to obtain the aggregated form. Under anesthetization, peptides (1 μL = 5 μg) were injected into the bilateral hippocampuses, with stereotaxic coordinates from the bregma being, in mm, A -3, L/R-2.0, and V 3.5. After analepsia, berberine chloride (50 mg/kg) (Sigma, St. Louis, MO. USA) was administered intragastricly once daily for 14 days. All experimental animals were overseen and approved by the Animal Care and Use Committee of Sun Yet-Sen University before and during experiments.

### Morris water maze test

White-colored water was poured into a circular pool (diameter, 120 cm; height, 28 cm), and a white platform (diameter, 8.3 cm) was placed 1.5 cm below the water level in the middle of a fixed quadrant. The water temperature was adjusted to 25 ± 1°C. Memory-acquisition trials (training) were performed four times daily for 7 days to reach a steady state of escape latency. At 1 h after intragastric administration of berberine chloride, the rats were allowed to swim freely for 120 s and were left for an additional 30 s on the platform. The intertribal interval during four trials was 75 min. Start positions set at each limit between quadrants, were randomly selected for each animal. Rats failing to find the platform were placed on the platform manually. Memory-retention tests were performed at the seventh day after the last training session. The platform was removed, and each rat was allowed a free 120 s swim. The number of crossings over a point where the platform had been was counted by replay using a video recorder. Data are presented as mean ± SD.

### Immunohistochemistry

Animals were perfused through the heart under deep anaesthesia (chloral hydrate 0.35 mL/100 g) with 100 mL of phosphate buffer saline (PBS) containing 10 U/mL heparin followed by 150–200 mL of 4% paraformaldehyde in PBS, PH 7.4. Brains were removed and then immersed in PBS containing sucrose, PH 7.4, first in 20% sucrose for 24 h and then in 30% sucrose until sunk (2–5 days). 8 μm sections were cut on a cryostat and mounted in polylysine -coated slices. The number of IL-1β and iNOS positive cells around the point of injection were analyzed immunohistochemically using an antibody against IL-1β (sc-7784, dilution 1:50, Santa Cruz Biotechnology Inc., USA) and anibody against iNOS (SC-651, dilution 1:50, Santa Cruz Biotechnology, Inc.USA.). For the visualization under light microscope, incubation with biotinylated rabbit/mouse anti-rat IgG (Dako Cytomation Inc., USA) and the immunoreaction was visualized using diaminobenzidine. Counting of IL-1β and iNOS -expressing cells was done by an observer blind to the treatment status of the rats based on the visibility of a cell soma in 8 μm-thick coronal sections in 400 × fields by Olysia Bioreport software (Olympus company, Japan). Four slides around the injection point were evaluated per animal. Data are presented as mean ± SD.

### Real time PCR

Expressions of genes were further confirmed by real time PCR. Primers were designed and synthesized by Takara biotechnology Co., Ltd (Dalian, China). Total RNA was isolated from hippocampus tissues using RNAiso reagent (Takara biotechnology Co., Ltd, Dalian, China). First-strand cDNA was synthesized from 0.5 μg of total cellular RNA with randoms 6 mers with the ExScript™ RT Reagents Kit (Takara biotechnology Co., Ltd, Dalian, China). After incubation for 15 mins at 42°C, the RT mixture was incubated at 95°C for 2 min to inactivate the reverse transcriptase. Real time PCR cycles were carried out for amplification of IL-1β, iNOS and β-actin cDNA using a thermal cycler (MJ Research company, DFC-3200, USA). The primers sequences for IL-1β were 5'- GCT GTG GCA GCT ACC TAT GTC TTG -3' (sense) and 5'- AGG TCG TCA TCA TCC CAC GAG -3' (antisense). The primer sequences for iNOS were 5'- CTC ACT GTG GCT GTG GTC ACC TA -3' (sense) and 5'- GGG TCT TCG GGC TTC AGG TTA -3' (antisense). The primer sequences for β-actin were 5'- TGA CAG G TG CAG AAG GAG A -3'(sense) and 5'- TAG AGC CAC CAA TCC ACA CA-3'(antisense). Real time PCR was then carried out using 2 μL cDNA in a final reaction volume 20 μL using SYBR Premix Ex Taq™ (Takara biotechnology Co., Ltd, Dalian, China). The PCR cycling program was set for 1 cycle of pre-denaturation at 95°C for 10 s, and then 39 cycles at 95°C for 5 s, 60°C for 20 s, 81°C plate reading for 3 s, melting curve from 55°C to 95°C read every 0.2°C hold for 1 s between reads. The relative copies of IL-1β mRNA and iNOS mRNA on the internal control β-actin are arbitrary units.

### Statistical analysis

All of the data were expressed as mean ± SD and the analysis was carried out using the one way analysis of variance(ANOVA) or two-way RM ANOVA. Values of P < 0.05 were considered statistically significant.

## Abbreviations

AD: Alzheimer's disease

PCR: polymerase chain reaction

IL-1β: Interleukin-1β

iNOS : Inducible nitric oxide synthase

IGF-1:insulin-like growth factor-1

NO: nitric oxide

PBS: phosphate buffer saline

## Authors' contributions

FZ and CQ participated in the design of the study. FZ carried out the spatial memory test, immunohistochemistry, real time PCR, performed the statistical analysis and wrote the manuscript. All authors read and approved the final manuscript.
